# Rooibos (*Aspalathus linearis*) Genome Size Estimation Using Flow Cytometry and K-Mer Analyses

**DOI:** 10.3390/plants9020270

**Published:** 2020-02-18

**Authors:** Yamkela Mgwatyu, Allison Anne Stander, Stephan Ferreira, Wesley Williams, Uljana Hesse

**Affiliations:** 1 South African National Bioinformatics Institute (SANBI), University of the Western Cape, Robert Sobukwe Road, Bellville 7535, South Africa; yamkelamgwatyu@gmail.com; 2 Department of Biotechnology, University of the Western Cape, Robert Sobukwe Road, Bellville 7535, South Africa; allison.stander@outlook.com (A.A.S.); wesleywt@gmail.com (W.W.); 3 WestCape Biotech, Portion 26 of Farm 27, R304, Koelenhof, Stellenbosh 7605, South Africa; S.Ferreira@genetwister.nl

**Keywords:** Rooibos, *Aspalathus linearis*, flow cytometry, k-mer analysis, genome size, ITS region

## Abstract

Plant genomes provide information on biosynthetic pathways involved in the production of industrially relevant compounds. Genome size estimates are essential for the initiation of genome projects. The genome size of rooibos (*Aspalathus linearis* species complex) was estimated using DAPI flow cytometry and k-mer analyses. For flow cytometry, a suitable nuclei isolation buffer, plant tissue and a transport medium for rooibos ecotype samples collected from distant locations were identified. When using radicles from commercial rooibos seedlings, Woody Plant Buffer and *Vicia faba* as an internal standard, the flow cytometry-estimated genome size of rooibos was 1.24 ± 0.01 Gbp. The estimates for eight wild rooibos growth types did not deviate significantly from this value. K-mer analysis was performed using Illumina paired-end sequencing data from one commercial rooibos genotype. For biocomputational estimation of the genome size, four k-mer analysis methods were investigated: A standard formula and three popular programs (BBNorm, GenomeScope, and FindGSE). GenomeScope estimates were strongly affected by parameter settings, specifically CovMax. When using the complete k-mer frequency histogram (up to 9 × 10^5^), the programs did not deviate significantly, estimating an average rooibos genome size of 1.03 ± 0.04 Gbp. Differences between the flow cytometry and biocomputational estimates are discussed.

## 1. Introduction

Rooibos (*Aspalathus linearis* (Burm.F.) Dahlg.) is a leguminous shrub native to the Cape Floristic Region of South Africa [1]. It is produced commercially to make the well-known beverage, rooibos herbal tea, which has reached both local and international acclaim [2,3]. Rooibos is also increasingly recognized as a potential phytopharmaceutical: the species produces a wide range of phenolic compounds, which are associated with diverse medicinal properties of the plant (including anti-diabetic, cardioprotective, antispasmodic, anti-aging effects; reviewed in [4]). Of particular interest are rare and unique compounds, such as the dihydrochalcones aspalathin and nothofagin, and the phenylpropeonic acid glycoside PPAG, which show preventive potentials against the metabolic syndrome [5].

Commercial rooibos farming is confined to the natural habitat of the species—the larger Cederberg Mountain region of South Africa, spanning from Nieuwoudtville (Northern Cape) in the North to Piketberg (Western Cape) in the South [2]. Small-scale farmers in the Suid Bokkeveld and Wupperthal areas also harvest wild rooibos, which is marketed as an organic and fair-trade certified product [2,6,7]. Growth of the rooibos industry is hampered by the shortage of good-quality plant material, which lead to the initiation of a genetic improvement program for rooibos [8]. Desirable traits include enhanced drought and biotic stress tolerance, increased biomass production and stable production of selected phytochemicals. Rooibos genome data could provide valuable information on genes and biosynthetic pathways associated with desirable phenotypic traits, which in turn could serve as biomarkers for targeted plant selection and rooibos breeding. Prerequisite for successful genome sequencing is knowledge on genome characteristics of the species, specifically genome size, ploidy levels, and chromosome numbers.

Currently, the species *Aspalathus linearis* comprises several morphologically distinct growth types, including prostrate shrublets (Southern and Northern sprouters); more or less densely branched shrubs (Nardouwsberg type, Grey sprouter, Nieuwoudtville sprouter, Wupperthal type); and erect, slender bushes (Red type, Black type, Tree type) as described by [2,9,10]. These growth types vary in plant height, shoot numbers, reproductive morphology, niche adaptation mechanisms (drought and fire) and biochemical profiles [9,10,11,12]. The observed diversity is hypothesized to have emerged through geographic isolation and adaptation to differing environmental conditions, which may have led to accumulation of various mutations and the observed phenotypic plasticity [12]. The commercially cultivated rooibos plants (aka Nortier type or Rocklands type) descended from successively selected wild “Red type” plants, originally collected from the northern parts of the Cederberg Mountains and the Pakhuis Pass areas [10]. As established by Dahlgren in 1968, the cultivated rooibos plants are diploid and have a chromosome number of 2*n* = 18. This is likely true for all growth types of the *A. linearis* species complex, since these characteristics are conserved in the *Aspalathus* genus: All of the 133 *Aspalathus* species investigated to date were found to be diploid, and 85% of these species have a base chromosome number of 9 [13]. Genome sizes have not yet been established for any of the species in the *Aspalathus* genus.

Genome size refers to the amount of DNA in the unreplicated gametic nucleus [14], which can be measured either in picograms (pg) or mega base pairs (Mbp, with 1 pg = 978 Mbp). It is an essential parameter in genome sequencing projects, as it determines the budgeting for planned sequencing depths and provides a ballpark number to estimate completeness of genome assemblies. Flow cytometry represents a cost-effective, relatively accurate and rapid laboratorial technique for plant genome size estimation. It involves estimation of DNA amounts based on the staining of intact nuclei with a fluorochrome that binds to the DNA in a quantitative manner. However, the method must be adapted for each plant species, since high concentrations of phenolic compounds, specifically in woody plants (such as rooibos), may cause stoichiometric errors. Genome sizes can also be estimated biocomputationally through k-mer analysis of Illumina sequencing data. A number of biocomputational tools for the generation of k-mer frequency histograms (e.g., KAT [15], Jellyfish [16]) and k-mer based genome size estimation (BBNorm [17], Genomescope [18,19], FindGSE [20]) have been developed.

In this study, we used flow cytometry to estimate the genome sizes of eight different growth types from the *A. linearis* species complex. We tested four nuclei isolation buffers, three plant tissues and four transport media for rooibos leaf material collected from distant locations. Based on the genome size estimates for commercial rooibos plants, we generated short-read Illumina sequencing data from one commercial rooibos genotype. We used this data to estimate the rooibos genome size using k-mer analyses and investigated the effects of read coverage, quality filtering of the reads, k-mer size and maximum k-mer coverage.

## 2. Results

### 2.1. Flow Cytometry-Method Adaptation

**Figure 1:**
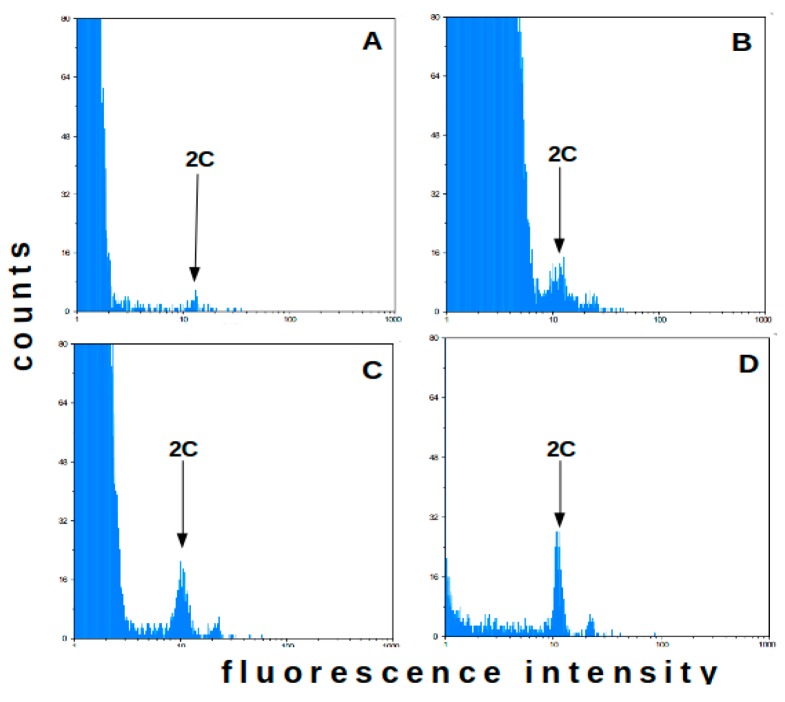
Flow cytometry histograms for *A. linearis* leaves from 3 weeks-old commercial rooibos seedlings using Partec buffer (**A**), LBO1 10X Triton X-100 buffer (**B**), LBO1 5X Triton X-100 (**C**), and Woody Plant Buffer (**D**). Sample 2C (GO/G1 phase) peaks are shown (*n* = 10).

For method adaptation, commercial rooibos seedlings (Red type) were used. Four nuclei isolation buffers were tested for their suitability in flow cytometry analyses to estimate the rooibos genome size: Partec buffer, LB01 with 5x Triton X-100 (LB01-5x), LB01 with 10x Triton X-100 (LB01-10x) and Woody Plant Buffer (WPB). Representative histograms for each buffer are shown in Figure 1 (*n* = 10). When evaluating the 2C (G0/G1) peak of *A. linearis*, no distinct peak was observed for the commercial Partec buffer. Peaks could be distinguished when using LB01-5x and LB01-10x buffers in most plant samples, but failure rates were high (30% and 60%, respectively). Best results were obtained with WPB, where high-resolution peaks and low debris background were obtained for all replicates.

**Figure 2:**
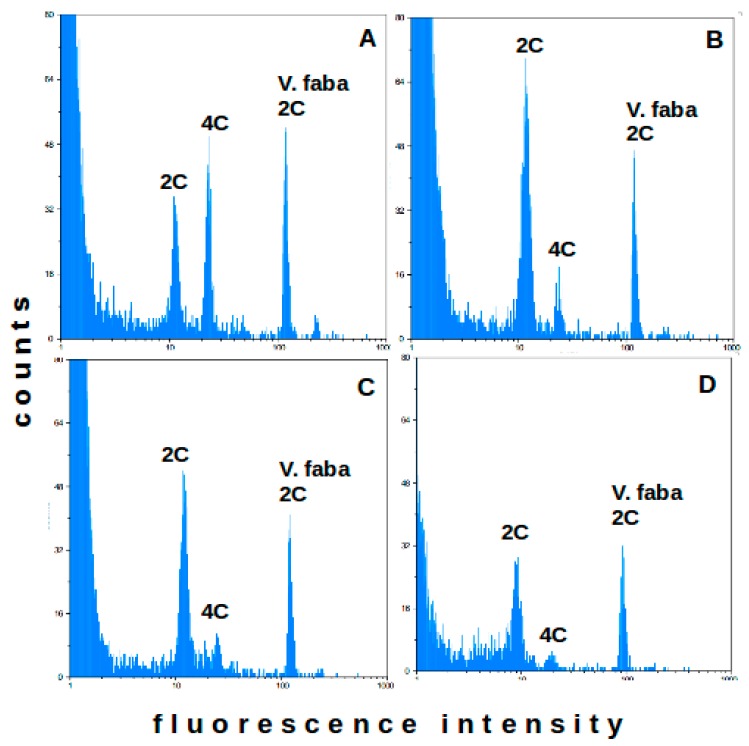
Flow cytometry histograms of rooibos radicles (**A**) and cotyledons (**B**), as well as fresh leaves (**C**) and silica-dried leaves (**D**) from two-months-old commercial rooibos seedlings. 2C (G0/G1 phase), 4C (G2 phase) and reference standard (*Vicia faba;* 2C=26.66 pg) 2C peaks are shown. (*n* = 10).

Subsequently, WPB was used to isolate and stain nuclei from radicles and cotyledons, as well as fresh and dried leaves from two-months-old commercial rooibos seedlings (Figure 2). In this test, higher debris backgrounds were observed. Nonetheless, all freshly harvested plant tissues generated high resolution histograms with distinct peaks (Figure 2A–C), which allowed genome size estimations using *Vicia faba* (2C = 26.66 pg) as an internal reference standard. Plant tissue type significantly affected the estimation of the 2C DNA contents. With 2.54 pg, radicles had significantly lower values (*p* < 0.05) than cotyledons (2.64 pg) and leaves (2.69 pg). We also noted that the 4C peak was more prominent in the radicle histograms than in the cotyledon and leaf histograms. The calculated 1C genome sizes for these tissues equaled 1.24 ± 0.01 Gbp, 1.29 ± 0.02 Gbp, and 1.31 ± 0.02 Gbp, respectively.

For best results, flow cytometry analyses are typically conducted on fresh plant material. When samples are collected in remote regions, a transport medium has to be identified that would preserve the plant material (in our case for 4–5 days, the typical duration of the sample trips). Four preservation media were tested: sterilized water, 5% glycerol solution, 10% glycerol solution and silica-gel crystals. All samples maintained in liquid media turned brown within hours of collection. Preliminary tests had shown that brown rooibos plant tissues did not yield peaks in the flow cytometry histograms, and flow cytometry analyses failed for most samples from these media. Silica-gel crystals kept plant tissues green for the entire specified period. The 2C peaks in the histograms for silica-dried leaves were smaller and broader than those obtained for fresh plant material, but were still suitable for genome size estimation (Figure 2D). With 1.26 ± 0.05 Gbp, the average genome size estimate for dried rooibos leaves was comparable to the estimates obtained with fresh plant material. Silica gel was therefore used to preserve plant samples collected from commercial and wild rooibos ecotypes at distant sampling locations.

### 2.2. Flow Cytometry—Analysis of Field Plants

A total of 82 commercial and wild *A. linearis* plants representing nine rooibos growth types were randomly sampled from 17 populations located in the broader Clanwilliam, Wupperthal, and Nieuwoudtville regions (Figure S1). To verify morphology-based taxonomic classification of the rooibos field plants, we sequenced the ITS region and aligned these sequences to the 15 ITS sequences from 14 *Aspalathus* species currently available at NCBI (including 2 sequences for *A. linearis*). The alignment, showing the results for two plants per growth type, comprised 711 nucleotides (Figure S2). Our results showed that (1) all ITS sequences from our dataset were identical, with highest similarity to previously published *A. linearis* ITS sequences (99.9% sequence identity to EU347722); (2) the single nucleotide polymorphism in position 567 of the *A. linearis* sequence EU347722 and the polymorphic site in position 109–117 of the *A. linearis* sequence AJ744951 are not conserved in the *A. linearis* species complex; and (3) three conserved single nucleotide polymorphisms in the sequenced ITS region (positions 537, 695, and 697) distinguish the *A. linearis* and *A. pendula* species from the other *Aspalathus* species. These results are in line with previous phylogenetic analyses that place *A. linearis* and *A. pendula* into a distinct clade within the *Aspalathus* genus [21]. Therefore, our ITS analyses supported the morphology-based taxonomic classification of the sampled plants into the *A. linearis* species complex.

**Figure 3:**
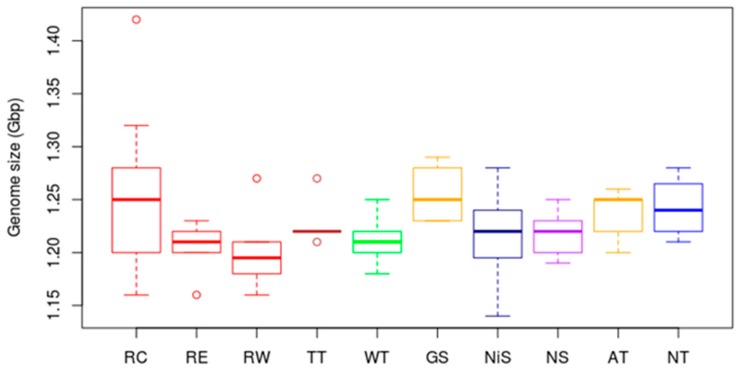
Flow cytometry genome size estimates for different rooibos growth types using silica-dried leaf samples (RC = Red Commercial (*n* = 10), RE = Red Escaped (*n* = 5), RW = Red Wild (*n* = 6), WT = Wupperthal Type (*n* = 9), TT = Tree Type (*n* = 5), GS = Grey Sprouter (*n* = 5), NiS=Nieuwoudtville Sprouter (*n* = 11), NS = Northern Sprouter (*n* = 5), AT = Algeria Type (*n* = 5), NT = Nardouwsberg Type (*n* = 4)).

Flow cytometry analyses of silica-dried leaf samples were successful for most plants, but failed for all five Black type plants, and the ten plants from one of the two sampled Grey sprouter populations. Figure 3 shows the flow cytometry genome size estimates for silica-dried leaf samples from rooibos plants collected in different locations. The dried leaf samples from commercial field plants (Red type, RC) showed highest variability in genome size estimates, ranging from 1.16 Gbp to 1.42 Gbp. Interestingly, the mean genome size estimate and the standard deviation for these samples (1.26 ± 0.08 Gbp) mirrored the results obtained for the dried leaf samples in the seedling test (1.26 ± 0.05 Gbp). The samples from two populations of wild-growing Red type plants, including one escaped commercial (RE) and one wild Red type (RW) population, had an average genome size estimate of 1.20 Gbp, showing low deviations from the sample means. The average genome size estimates for all other rooibos growth types ranged from 1.21 Gbp to 1.26 Gbp, i.e., did not differ substantially from the genome size estimates of the Red type plants.

### 2.3. Illumina Sequencing

The paired-end 300 bp insert Nextera library was sequenced using the MiSeq and the HiSeq 2500 Illumina sequencing platforms, resulting in 0.6 billion and 1.9 billion read pairs, respectively (Table 1). Average read length of the raw data after adapter removal was 119 bp. Quality filtering (trimming of bases below Phred score of 20, and removal of reads shorter than 50 bp) did not substantially reduce the dataset: 2.4 billion read pairs were retained and average read length was 120 bp. Based on the flow cytometry estimates of the rooibos genome size for radicle samples (1.24 ± 0.01 Gbp), the total genome coverage from the raw and quality processed data amounted to 245x and 234x, respectively. The MiSeq dataset yielded 58x genome coverage (both raw, and quality processed data).

### 2.4. K-Mer Analysis

For computational genome size estimation using k-mer analysis, we investigated four methods, including the most recent dedicated programs (BBNorm, GenomeScope, and FindGSE) and the often-used formula for genome size calculations derived from equations introduced by the M.S. Waterman group [22,23]. For each program, we investigated the effects of (1) MiSeq sequence subset vs complete dataset (MiSeq and HiSeq data), (2) k-mer size, and (3) raw vs quality processed data. The results are provided in Table 2. Our analyses showed that the performance of GenomeSope (both v1 and v2) was strongly affected by parameter settings: the rooibos genome size estimates varied from 0.51 Gbp to 1.01 Gbp. The most influential parameter was the cutoff threshold for maximum k-mer coverage (CovMax). Increasing the threshold from 1k (default setting of GenomeScope v1) to 10k or 900k resulted in average increases of genome size estimates by 0.14 Gbp and 0.33 Gbp, respectively. Genome size estimates also increased with increasing k-mer size. The differences were higher at the lower CovMax settings, ranging from 0.17 Gbp at 1k, 0.11 Gbp at 10k, to 0.01 Gbp at 900k. For GenomeScope, the effects of using the MiSeq subsets vs complete datasets and raw vs quality processed data were small (<0.10 Gbp). FindGSE predicted a rooibos genome size of 1.06 ± 0.03 Gbp (averaged over all tested parameters). With this program, the differences between the MiSeq subset and corresponding values in the complete dataset were small (ranging from 0.01 Gb to 0.09 Gb). Increasing k-mer size only marginally increased genome size estimates (max by 0.04 Gbp), and differences between raw and quality processed datasets were also small (max 0.04 Gbp). BBNorm estimated a rooibos genome size of 1.08 ± 0.03 Gbp. The differences between the MiSeq subset and the complete dataset were minimal (varying from 0.00 to 0.06 Gbp, with higher estimates obtained for the complete dataset). An increase in k-mer size increased genome size estimates by only 0.05 Gbp. Differences between quality processed and raw datasets amounted to maximum 0.04 Gbp.

When using the formula, the rooibos genome size estimate amounted to 1.03 ± 0.04 Gbp. The effects of dataset size, k-mer size and data quality were also small (at most 0.08 Gbp, 0.05 Gbp, and 0.04 Gbp, respectively). When using the complete histogram profile (max k-mer coverage 900,000×) and averaging the values across all four methods and parameter settings, the computational estimate of the rooibos genome size was 1.03 ± 0.05 Gbp.

## 3. Discussion

Rooibos produces a range of compounds that are known to interfere with flow cytometry analyses. It is rich in phenolic compounds (e.g., PPAG and aspalathin, [24]) that bind to the DNA, reducing fluorochrome accessibility and stoichiometric staining of the DNA [25,26]. Aspalathin alone can contribute up to 13.5% to the leaf dry weight (2%–6% on average), and total polyphenols can make up to 30% of the plant dry weight [27]. Buffer selection therefore focused on those previously found suitable for flow cytometry analyses of recalcitrant and/or woody plant species. The Partec buffer, though successfully used on woody and non-woody plant species rich in secondary metabolites, including coconut [28], honeybush [29], *Lupinus* sp. [30], and *Brassica* sp. [31], did not produce peaks with rooibos leaf samples. Here, high polyphenol concentrations in the rooibos leaf samples may have inhibited nuclei isolation. Addition of compounds such as PVP, which bind polyphenols, were found to be essential for successful application of the Partec buffer in flow cytometry analyses of aromatic herbs of the genus *Zingiberacea* [32] and chamomile [33]. The same may have been true for LB01, which failed or generated low resolution peaks with most rooibos samples. Woody Plant Buffer (WPB) had been developed specifically for flow cytometry analyses of woody plant species [34]. It contains both PVP and DTT (a reducing agent), which counteract the effects of staining inhibitors, and was found suitable for a wide variety of recalcitrant plant species [25,26,34]. When using rooibos leaves, it was the only buffer that consistently generated peaks of sufficient quality to estimate the rooibos genome size.

To verify, if plant tissue type affects peak quality and genome size estimates, WPB was also tested on rooibos radicles and cotyledons. *Vicia faba* was found to be a suitable reference standard for rooibos genome size estimation, because its large genome size (2C = 26.66 pg) ensured clear separation of the fluorescent signals originating from rooibos and reference standard nuclei, respectively. Best flow cytometry results were obtained for radicles, where CV% values were consistently below 5%. The prominent 4C peak observed in the radicle samples likely reflects higher cell division in the rapidly growing root tissues as compared to the cotyledon and leaf tissues [35,36]. Interestingly, the average genome size estimates for radicles (1.24 ± 0.01 Gbp) were marginally but consistently smaller than those for cotyledons and leaves (by 0.05 Gbp and 0.08 Gbp, respectively; both *p* < 5%). This may reflect higher amounts of polyphenols in the cotyledon and leaf tissues. Analyses with different plant species [37] showed that plant tissues richer in polyphenols yielded higher flow cytometry genome size estimates than plant tissues with low polyphenol contents. In rooibos, aspalathin concentrations have been shown to be higher in leaves than in stems [38], but comparative analyses for seedling tissues are outstanding.

Typically, fresh leaf material is the material of choice for flow cytometry analyses. For rooibos, once harvested, leaves turn red-brown in a very short period of time, which is mostly due to the degradation of aspalathin [39]. Preliminary flow cytometry tests showed that these tissues do not yield peaks in the histograms. For commercial rooibos plants, seeds can be purchased and seedling populations providing fresh plant material can be established at the research site. For wild rooibos plants seeds are not available commercially, and seed collection is exceptionally difficult: The black rooibos seeds are as small as ant eggs and difficult to distinguish from soil particles. Moreover, seed treatment (scarification) and germination methods would have to be adapted, since germination is likely to be affected by seed ripeness. To investigate genome sizes of the different rooibos growth types that grow in remote locations, a transport media had to be identified that would preserve plant tissues for at least four days (the typical duration of the sampling trips). Our analyses showed that for most rooibos plants silica gel crystals were suitable for this purpose: the plant tissue was still green and yielded distinct peaks in flow cytometry histograms. However, intensive chopping was necessary to obtain sufficient numbers of intact nuclei. Despite all efforts, flow cytometry analyses failed for the Black type plant samples and all 10 plant samples from one Grey sprouter population. These plants had very tough leaves and were difficult to chop, yielding insufficient numbers of intact nuclei. For these plants, further method adaptation is required. For silica-dried plant samples from commercial rooibos seedlings and field plants, we noted that the DNA content estimates were more variable (stdev 0.08 and 0.05 Gbp, respectively) than with any of the fresh rooibos samples (max stdev 0.02). This is likely due to differences in the chemical profiles of the plants (which grew in three distant locations and were therefore exposed to different environmental conditions) and the degree of polyphenol degradation in the silica-dried leaf material. The standard deviations for wild rooibos growth types where only one population was sampled, were smaller (0.02–0.04 Gbp). The mean genome size estimates for the wild rooibos growth types were within the range obtained for commercial rooibos samples. Therefore, the morphological differences between the rooibos growth types are not likely to be associated with variations in ploidy levels or significant genome expansion events.

For genome sequencing, the genome size estimate obtained for the radicle samples (1.24 ± 0.01 Gbp) was used, since these samples generated the best quality peaks, and the higher estimates for cotyledon and leaf samples are likely associated with higher polyphenol contents in the plant material. Subsequently, k-mer histograms generated from the Illumina sequencing data were used to estimate the rooibos genome size biocomputationally. GenomeScope was the only program that stood out, as genome size estimates were strongly affected by parameter settings, specifically k-mer coverage thresholds (CovMax). At lower CovMax thresholds, highly covered k-mers, mostly representing repeats, are excluded from the analyses, which may substantially reduce genome size estimates. In GenomeScope v1, the default for CovMax is set to 1k. This low default threshold may explain why, in studies with vanilla [40], cane toad [41], and pacific oyster [18,42], k-mer based GenomeScope estimates of genome sizes were only half of those obtained by flow cytometry analyses and considerably smaller than those obtained after genome assembly. For rooibos, GenomeScope estimates differed between 0.51 and 1.01 Gbp, indicating that with this program parameter settings must be adjusted, based on reasonable assumptions on the genome under investigation (repeat content, data preprocessing). When using the complete k-mer histogram (up to a k-mer coverage of 900,000×), the investigated programs and parameter settings did not differ substantially in genome size estimates, yielding an average genome size of 1.03 ± 0.05 Gbp. This value is approximately 0.2 Gbp below the flow cytometry value obtained for rooibos radicles. Similar discrepancies have been reported for European eel [43], and even for the model plant *Arabidopsis thaliana* [20] and were argued by the authors to be associated with the interference of chemical compounds in the stoichiometric DNA content measurements in flow cytometry analyses. In our study, the equipment related restrictions in the choice of the fluorescent stain (DAPI) may have also led to higher flow cytometry genome size estimates: DAPI predominantly binds to AT-rich regions in the DNA, and plant genomes are known to be riddled with AT-rich repeats. We therefore conclude that the k-mer based rooibos genome size estimate is closer to the true value.

## 4. Materials and Methods

### 4.1. Plant Material

Pre-treated commercial rooibos seeds (scarified and coated with pesticide) were provided by Rooibos Ltd. (Clanwilliam, Western Cape, South Africa). These seeds were collected in 2017 from commercial rooibos plants from diverse farms in the Cederberg Mountains, and therefore represent a mixture of commercial rooibos genotypes.

A study population of commercial rooibos plants was established at the University of the Western Cape as follows: First, 200 rooibos seeds were germinated on moist filter paper in glass Petri dishes at room temperature and ambient light for two days. Thereafter, 90 seedlings with a distinguishable radicle were transferred to pots (9 cm diameter) containing non-pasteurized Clanwilliam soil (3 seedlings per pot), and maintained in the greenhouse at 26 °C. The seedlings were watered daily for 7 days, and then at 2-to-4 day intervals. Leaves and twigs from two months old plants were used in experiments to identify an appropriate buffer for flow cytometry analyses and to find a suitable transport/storage media for rooibos leaf samples from distant locations.

For flow cytometry analyses of cotyledons and radicles, 40 rooibos seeds were surface sterilized by soaking for 20 min in 6% sodium hypochlorite (Kimix, Cape Town, South Africa) and rinsing five times with sterile water. The seeds were then germinated on PDA (containing 30 mg/mL penicillin and 25 mg/mL streptomycin) at room temperature in the dark (5 seeds per plate). To exclude contaminations, all healthy-looking 5 day old seedlings were transferred to new PDA plates (1 seedling/plate) and maintained at RT in light for 3 days. For flow cytometry analyses of cotyledons and radicles, 10 healthy seedlings were analyzed, respectively using woody plant buffer (see below).

To identify an appropriate media for transport of rooibos samples from distant locations that would maintain plant material suitable for flow cytometry analyses, four media were tested: (1) Distilled water, (2) 5% glycerol solution, (3) 10% glycerol solution, and (4) 3.5 mm silica gel beads (Lasec). For each treatment, four twigs with green leaves from ten different rooibos plants of the study population were placed into 50 mL falcon tubes (1 tube per plant) and completely submerged in the respective media. The tubes with silica gel beads were maintained at ambient temperatures, and the tubes with liquid media at 4 °C. After four days, the samples were visually examined and analyzed by flow cytometry using woody plant buffer (see below).

For field plant analyses, commercial plants from three rooibos farms in distant locations were sampled. Wild *A. linearis* populations, representing nine different rooibos growth types, were identified in the larger Clanwilliam, Wupperthal, and Nieuwoudtville regions with the help of local rooibos farmers (Figure S1). Taxonomic classification of the wild rooibos plants was based on morphological characterization of the plants, supportive information from the farmers and growth type verification by the South African botanist B.E. van Wyk [10]. In addition, we sequenced the ITS region from all sampled field plants (see below). Depending on the size of the population, five to ten plants were sampled per population (Table S1). For DNA analyses, leaf material was flash-frozen in the field using liquid nitrogen, transported on dry ice and maintained at −80 °C. For flow cytometry analyses, two twigs with healthy, green leaves were cut from four sides of the plants (eight twigs in total), transferred to 50 mL Falcon tubes, completely covered with 3.5 mm silica gel beads and transported at ambient temperatures in a dry container to the study site. Flow cytometry analyses were conducted within four days of collection on three different leaf samples per plant using woody plant buffer (see below).

### 4.2. Flow Cytometry Analyses

Identification of a suitable buffer for flow cytometry analyses of rooibos plant material was conducted using 10 rooibos plants from the study population. Four nuclei isolation buffers were tested: Partec buffer (CyStain^®^ UV Precise P Nuclei isolation buffer, CyStain^®^ UV Precise Staining Buffer (contains DAPI), *v*/*v* = 1/4), LB01 with 10x Triton X-100 (LB01-10x: 15 mM TRIS, 2 mM Na_2_EDTA, 0.5 mM spermine, 80 mM KCl, 20 mM NaCl, 10x Triton X-100, 1 mg/mL DAPI, pH 7.5; [43]), LB01 with 5x Triton X-100 (LB01-5x) [44] and Woody Plant Buffer (WPB: 0.2 mM TRIS, 4 mM MgCl_2_.6H_2_O, 2 mM Na_2_EDTA.2H_2_O, 86 mM NaCl; 2 mM DTT, 1% (*w*/*v*) PVP-10, 1% (*v*/*v*) Triton X-100, 1 mg/mL DAPI, pH 7.5; [34]). From each plant, two to three rooibos leaves and the equivalent amount of reference standard leaves (*Vicia faba*) were co-chopped in a 55 mm Petri dish in the respective buffer using a sterile, sharp razor blade. For WPB and LB01, tissues were chopped in 1.6 mL of buffer augmented with 1 mg/mL 4,6 diamidino-2-phenylindole (DAPI) stain. For Partec buffer, plant material was chopped in 200 µL CyStain^®^ UV Precise P Nuclei isolation buffer and stained with 800 µL CyStain^®^ UV Precise Staining Buffer, containing DAPI. The suspensions were filtered through a 50 µm nylon filter to remove cell debris and incubated for 5 min at room temperature in a flow cytometry tube. The nuclei suspensions were analyzed at a wavelength of 375 nm using the CyFlow^®^ Space (Partec GmbH, Mϋnster, Germany) flow cytometer equipped with a UV laser diode of 16 mW. Results were statistically analyzed using FloMax 2.80 (Partec GmbH, Mϋnster, Germany), and regions of interest gated manually in a one-parameter histogram. Nuclear DNA content was calculated using the formula:

2C DNA content = (mean sample G1 peak)/(mean standard G1 peak) × standard 2C DNA content (pg)

Conversion of DNA mass (pg) to genome length (Gbp) was conducted using the equation:

Gbp = pg × 0.978

### 4.3. DNA Extraction, PCR Amplification and Sequencing

Rooibos genomic DNA was extracted using a modified protocol adapted from [45] and [46]. Young rooibos leaves were ground in liquid nitrogen using mortar and pestle to fine powder. For each gram of leaf tissue, 4ml of heated (65 °C) CTAB extraction solution (100 mM Tris-Cl pH 8, 20 mM EDTA pH 8, 1.4M NaCl, 2% *w*/*v* CTAB, 1% *w*/*v* PVP, 2% *v*/*v* 2-ME) and 0.5 mL CTAB/NaCl solution (10% *w*/*v* CTAB, 7M NaCl) was added. After vigorous mixing, the homogenate was transferred into a 15 mL falcon tube. An equal volume of CTAB extraction solution was added, the solution was mixed thoroughly by inversion and then incubated at 65 °C for 1 h. To maximize yields, the solution was vortexed every 15 min. Thereafter, an equal volume of Chloroform: Isoamyl alcohol (24:1) was added and mixed by inversion. The top aqueous phase was recovered after centrifugation for 5 min at 7500× *g*. A 1/10th volume of 65 °C CTAB/NaCl solution was added to the supernatant and mixed well by inversion. The top aqueous phase was recovered after centrifugation for 5 min at 7500× *g*. An equal volume of CTAB precipitation solution (50 mM Tris-Cl pH 8, 10 mM EDTA pH 8, 1% *w*/*v* CTAB) was then added and the solution was incubated at 37 °C overnight. After centrifugation for 5 min at 500× *g* and 4 °C, the supernatant was recovered. DNA was precipitated by adding 0.6 volumes of isopropanol to the supernatant, mixed well and centrifuged for 15 min at 7500× *g* at 4 °C. The pellet was then washed with 80% ethanol, dried, re-suspended in 200 μL high salt TE buffer (10 mM Tris-Cl pH 8, 0.1 mM EDTA pH 8, 1M NaCl) per gram starting material and stored at −20 °C.

The ITS region was PCR amplified using the 17SE and 26SE primers published by Sun et al. [47]. Each reaction tube contained 30–50 ng of template DNA, 10 µL of 5X High-Fidelity Phusion^®^ reaction buffer (Biolabs^®^ Inc, New England), 5 µL of dNTP stock solution (containing 2 mM of each dNTP), 2.5 µL of each primer (10 µM; Inqaba Biotec, Pretoria, South Africa), 0.5 µL of 2 u/mL Phusion^®^ High-Fidelity DNA polymerase (Biolabs^®^ Inc, France SASU, New England) and nuclease-free water (final volume 50µL). The amplification was conducted in a T100 thermal cycler (Bio-Rad, USA) under the following conditions: initial denaturation at 98 °C for 3 min, 34 cycles of denaturation at 98 °C for 10 s, annealing at 72 °C for 30 s, and extension at 72 °C for 30 s; and a final extension at 72 °C for 10 min. To verify the size and concentration of the PCR products, 8 µl of the PCR reactions were analyzed on a 1.2% (*w*/*v*) agarose gel using *PstI* as a molecular weight marker. For samples that showed no bands or weak bands on the agarose gel, the PCR reactions were repeated. The PCR products were purified using the QIAquick PCR Purification Kit (Qiagen, Germantown, Maryland, USA) and stored at −20 °C. Amplicons were sequenced bidirectionally (with 17SE and 26SE primers) by Macrogen Inc. (Netherlands, Europe) using a 96-capillary ABI 3730xl DNA analyzer (Applied Biosystems, Foster City, Carlifonia, USA) following standard procedures. Sequencing reads were quality trimmed using CLC Genomics Workbench version 7.0.1 (QIAGEN-Bioinformatics, Germantown, Maryland, USA) using a quality limit of 0.01, removing all bases below a quality score of 20. Quality processed reads (Appendix A) were aligned with published ITS sequences from the *Aspalathus* genus obtained from NCBI (AJ74495, EU347722, EU347738, EU347723, EU347736, EU347729, EU347726, EU347732, EU347734, EU347731, EU347728, EU347733, EU347727, EU347725, EU347730) using MAFFT [48] with default settings, and the alignments were manually edited using MEGA X [49].

DNA library preparation for genome sequencing was performed at the UKHC Genomics Core Laboratory (UK Chandler Hospital Lexington, KY 40536, USA). One paired-end 300 bp insert size library was prepared using the Nextera DNA Library Preparation kit (Illumina, San Diego, CA, USA), following the manufacturer’s instructions. Sequencing was performed using two flow cells (1 lane each) on the Illumina MiSeq platform (Illumina, San Diego, CA, USA) and one flow cell (six lanes) on the Illumina HiSeq 2500 platform (Illumina, San Diego, CA, USA), generating in total 2.5 billion read pairs of 120 bp length according to the manufacturers protocols. Adapter trimming and removal of the PhiX spike was performed by the service provider using Illumina’s bcl2fastq2 Conversion Software v2.20 (Illumina, San Diego, CA, USA). Subsequently, quality trimming was performed using Trimmomatic (v0.38) [50] to remove remaining adapter sequences, leading and trailing nucleotides with a Phred score below 20, and reads shorter than 50 bp (ILLUMINACLIP:NexteraPE-PE.fa:2:30:10 LEADING:20 TRAILING:20 MINLEN:50). Error correction was performed on filtered reads using Lighter (v1.1.1) [51] with k set to 31, α set to 0.1, and specifying a 2x genome coverage of 2.5 Gbp (the use of a 2x genome coverage was suggested by the author of the program due to high heterozygosity predicted for the rooibos genome, personal communications). Quality filtering was performed for each lane, and subsequently assessed using FastQC (v0.11.7) [52] and MultiQC (v1.7) [53].

### 4.4. K-Mer Analyses

The khist.sh script from BBNorm was used to produce k-mer frequency histograms for k-values equal to 19, 23, 27, and 47. Histlen was set to 900,000 (which calculates k-mer frequencies up to a coverage of 900,000×). Analyses were performed on the raw and quality processed datasets using first just the MiSeq data and subsequently the complete dataset (MiSeq plus HiSeq data). The 16 k-mer histograms were used to estimate the rooibos genome size using the programs GenomeScope (v1 and v2), FindGSE and BBNorm. Both GenomeScope versions were run using three maximum k-mer coverage settings: 1000×, 10,000×, and 900,000×. Read lengths were specified based on the FastQC outputs for the datasets (119 bp or 120 bp), and ploidy level was set to 2. FindGSE was run specifying the respective k-mer size and the expected k-mer coverage for the homozygous region (exp_hom). The value for exp_hom was selected by finding the k-mer coverage at the maximum height of the homozygous peak, and then using the k-mer coverage value two counts above that one, satisfying the requirement that fp < VALUE < 2*fp, where fp is the maximum frequency of the homozygous peak (as discussed on the FindGSE GitHub page: [54]). To estimate the genome size using BBNorm, the khist.sh script was run using the above parameter settings. The rooibos genome size was also estimated using the often-applied formula introduced by Waterman: genome size = total number of k-mers/k-mer coverage at the highest frequency of the homozygous peak (for rooibos—the second peak in the histograms). The formula was applied after excluding low frequency k-mers, which likely represent sequencing errors.

## 5. Conclusions

This study highlights that estimation of genome sizes in plants remains a challenging undertaking. Flow cytometry analysis may overestimate the values due to the effects of different plant compounds that affect binding of the stains, while values based on k-mer analysis may differ depending on parameter settings of the programs. For the latter, quality of the sequencing data may also play a role. With a genome size of 1.03–1.24 Gbp, *Aspalathus linearis* is one of the legume species with a relatively large genome (similar to *Lupinus angustifolius* [55] and *Pisum sativum* [56]). Differences in ploidy levels between the rooibos growth types can be all but ruled out.

## Figures and Tables

**Table 1:** Statistics for the Illumina sequencing data from one rooibos genotype.

	Raw Sequencing Data		Quality Processed Sequencing Data	
**MiSeq**	**# read pairs**	**read length (bp)**	**# read pairs**	**read length (bp)**
Flow cell 1	455045310	119	446988504	120
Flow cell 2	153531660	119	150862318	120
**HiSeq**	**# read pairs**	**read length (bp)**	**# read pairs**	**read length (bp)**
Lane 1	284728634	120	278778534	120
Lane 2	299563658	119	292564436	120
Lane 3	311852326	119	227020170	118
Lane 4	355362948	119	347367568	120
Lane 5	374476482	119	366378088	120
Lane 6	321173550	119	314166814	120

**Table 2:** Genome size estimates for one commercial rooibos genotype (in Gbp) using raw and quality processed Illumina sequencing data.

	K19		K23		K27		K47			
**MiSEQ (0.6 Billion read pairs)**	**Raw**	**QP**	**Raw**	**QP**	**Raw**	**QP**	**Raw**	**QP**	**Average**	**SD**
GenomeScope v1 (CovMax 1k)	0.60	0.60	0.64	0.64	0.67	0.67	0.74	0.75	0.66	0.06
GenomeScope v2 (CovMax 1k)	0.59	0.59	0.63	0.63	0.66	0.66	0.74	0.74	0.66	0.06
GenomeScope v1 (CovMax 10k)	0.76	0.76	0.79	0.79	0.81	0.81	0.84	0.85	0.80	0.03
GenomeScope v2 (CovMax 10k)	0.76	0.76	0.79	0.79	0.80	0.80	0.83	0.84	0.80	0.03
GenomeScope v1 (CovMax 900k)	0.97	0.97	0.97	0.97	0.97	0.97	0.95	0.95	0.97	0.01
GenomeScope v2 (CovMax 900k)	0.97	0.96	0.97	0.96	0.96	0.96	0.94	0.94	0.96	0.01
FindGSE	1.05	1.06	1.08	1.06	1.08	1.09	1.13	1.11	1.08	0.03
BBNorm	1.05	1.06	1.08	1.06	1.08	1.09	1.14	1.13	1.09	0.03
Formula	1.07	1.03	0.98	0.97	1.08	1.06	1.01	1.02	1.03	0.04
**MiSEQ + HiSEQ (1.9 Billion read pairs)**	**Raw**	**QP**	**Raw**	**QP**	**Raw**	**QP**	**Raw**	**QP**	**Average**	**SD**
GenomeScope v1 (CovMax 1k)	0.59	0.52	0.59	0.58	0.63	0.62	0.74	0.74	0.62	0.08
GenomeScope v2 (CovMax 1k)	0.58	0.51	0.58	0.56	0.61	0.60	0.73	0.73	0.61	0.08
GenomeScope v1 (CovMax 10k)	0.71	0.69	0.75	0.74	0.78	0.77	0.85	0.85	0.77	0.06
GenomeScope v2 (CovMax 10k)	0.70	0.69	0.74	0.72	0.76	0.75	0.84	0.83	0.76	0.06
GenomeScope v1 (CovMax 900k)	1.00	0.97	1.01	0.97	1.01	0.98	1.00	0.97	0.99	0.02
GenomeScope v2 (CovMax 900k)	1.00	0.96	0.99	0.96	0.99	0.96	0.99	0.96	0.98	0.02
FindGSE	1.01	1.04	1.01	1.04	1.01	1.05	1.04	1.06	1.03	0.02
BBNorm	1.07	1.04	1.08	1.04	1.08	1.05	1.11	1.07	1.07	0.02
Formula	1.07	1.02	1.06	1.01	1.06	1.02	1.00	0.97	1.03	0.03

K19–K47: k-mer sizes; QP: Quality Processed; CovMax: cutoff threshold for maximum k-mer coverage (varied for GenomeScope from 1k to 900k, 900k for FindGSE andBBNorm); SD: Standard Deviation.

## Supplementary Materials

The following are available online at https://www.mdpi.com/2223-7747/9/2/270/s1. Figure S1: Rooibos sample collection sites from different locations. Figure S2: ITS alignment of 20 sequences from *Aspalathus linearis* plants representing 10 different growth types (Appendix A) and 15 *Aspalathus* sp. sequences from NCBI. Table S1: Mean ± SD Genome sizes (in Gbp) of different rooibos growth types sampled from remote areas.

## Appendix A

The following sequences were submitted to GenBank, listing first the sequence identifier followed by the GenBank accession number in brackets: A.linearis_RC1 (MN736469), A.linearis_RC2 (MN736470), A.linearis_RE1 (MN736471), A.linearis_RE2 (MN736472), A.linearis_RW1 (MN736473), A.linearis_RW2 (MN736474), A.linearis_TT1 (MN736475), A.linearis_TT2 (MN736476), A.linearis_NT1 (MN736477), A.linearis_NT2 (MN736478), A.linearis_WT1 (MN736479), A.linearis_WT2 (MN736480), A.linearis_GS1 (MN736481), A.linearis_GS2 (MN736482), A.linearis_NiS1 (MN736483), A.linearis_NiS2 (MN736484), A.linearis_NS1 (MN736485), A.linearis_NS2 (MN736486), A.linearis_AT1 (MN736487), A.linearis_AT2 (MN736488)
