# Supplementary figures and images for: Rooibos (Aspalathus linearis) Genome Size Estimation Using Flow Cytometry and K-Mer Analyses
Source: Plants (Basel). 2020 Feb 18;9(2):270. doi: 10.3390/plants9020270 (PMC7076435; doi:10.3390/plants9020270)

## Slide 1
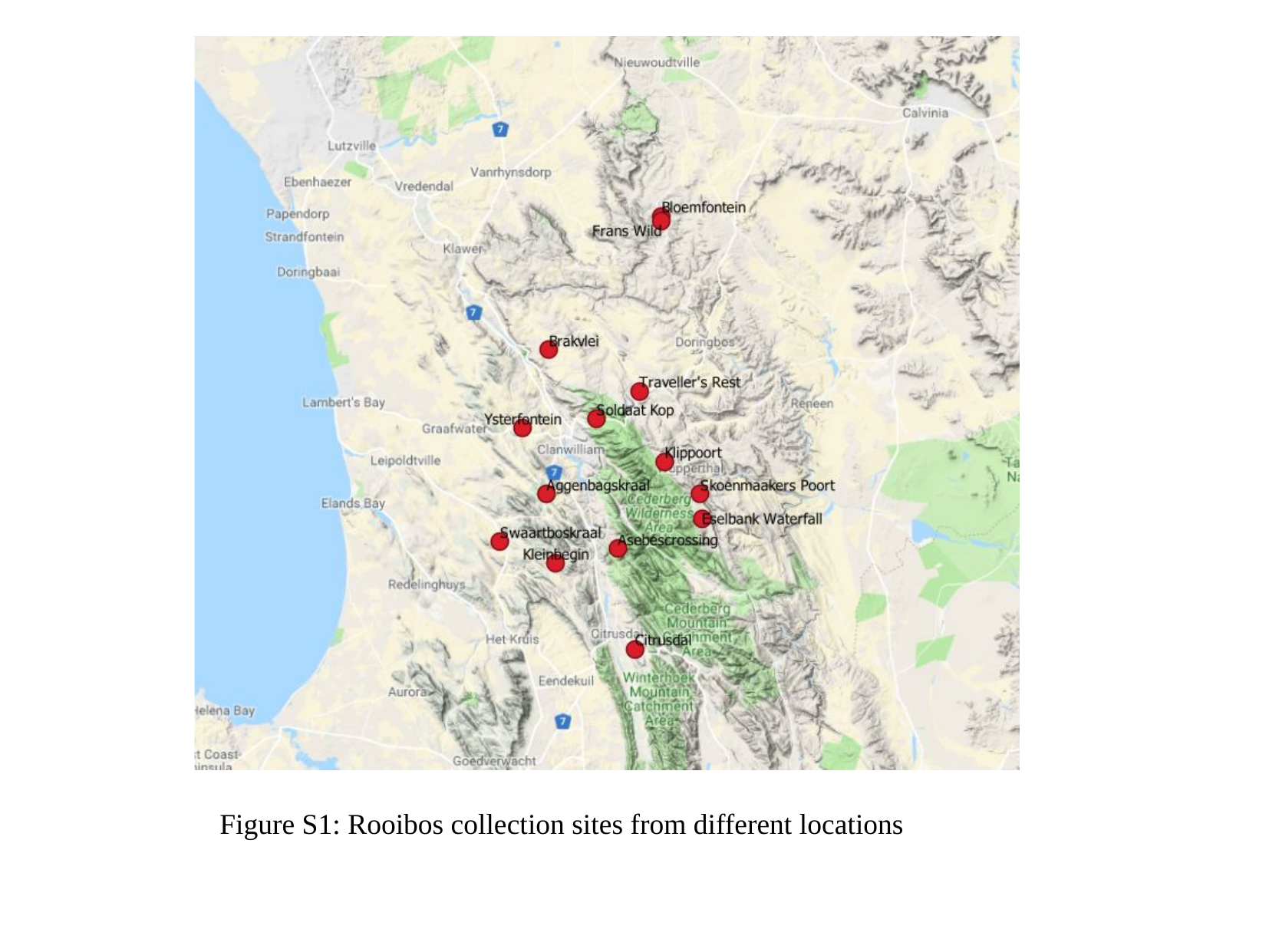

Figure S1: Rooibos collection sites from different locations

Supplement: Supplementary file 1 [file plants-09-00270-s001.zip › Supplementary_data/Figure_S1.pptx]
